# Sexual dimorphism of the ventral premammillary nucleus of the rat: stereological evaluation

**DOI:** 10.1186/s13293-025-00805-5

**Published:** 2025-12-12

**Authors:** Cássia Manuele Silva de Andrade, Fernando Vagner Lobo Ladd, Judney Cley Cavalcante

**Affiliations:** Laboratory of Neuroanatomy, Department of Morphology, Biosciences Center, Federal University of Rio Grande do Norte, 59078-900 Natal-RN, Brazil

**Keywords:** Hypothalamus, Stereology, Sexual dimorphism, Neuroanatomy, Sexual behavior, Aggressive behavior

## Abstract

**Background:**

The ventral premammillary nucleus (PMv), situated within the ventrobasal hypothalamus, is sensitive to steroid hormones and is involved in pheromone-responsive circuits. It maintains robust connections with sexually dimorphic nuclei both within and beyond the hypothalamus. Investigations over the past 15 years have suggested the role of the PMv in integrating environmental cues from conspecifics with internal states, thereby facilitating appropriate physiological and behavioral responses during reproductive and agonistic interactions. Neurochemical evidence indicates sexual dimorphism in the PMv of rats; however, comprehensive structural analyses are lacking.

**Methods:**

After perfusing and processing the brains of male and female rats during the estrus and diestrus phases, we applied stereological methodology in the PMv.

**Results:**

Males presented significantly greater neuronal volume and quantity than females did across both cycling phases. Neuronal volume in females was notably greater during estrus than during diestrus. However, no dimorphism was detected in overall volume, neuronal density, volume occupied by neurons, or neuropils.

**Conclusions:**

Given its role as a nexus between nutritional status and reproductive physiology, as well as its involvement in modulating agonistic behavior, including maternal aggression, structural disparities in the PMv between males and females may reflect divergent functional roles, contributing to sex-specific strategies in reproduction and aggression.

## Highlights

The ventral premammillary nucleus (PMv) is a hypothalamic nucleus known to influence both agonistic and reproductive behaviors.

We conducted a stereological analysis to explore the presence of sexual dimorphism within the PMv.

Our findings indicate that neither the overall volume nor the neuronal density of the PMv exhibits sexual dimorphism. Similarly, the volume occupied by neurons and the neuropil do not differ significantly between sexes.

However, male rats possess a greater number of neurons in the PMv, and these neurons exhibit a larger volume compared to female rats in both estrus and diestrus phases.

Female rats in estrus presented a greater neuronal somatic volume in the PMv than those in diestrus.

These observed sexual dimorphisms in the PMv, along with their hormonal modulation, may reflect the nucleus’s involvement in sex-specific behaviors, such as aggression and reproduction.

## Background

Sex differences in behavior, such as mating and aggression, are more obviously stereotyped. It is a consequence of a sexually differentiated nervous system [1]. The hypothalamus is the controlling center of motivated behaviors, and a small hypothalamic nucleus named the ventral premammillary nucleus (PMv) seems to be a key place for integrating conspecific environmental cues and the internal state, providing context for distinct social and sexually dimorphic behaviors, such as those mentioned above.

The PMv is a component of the accessory olfactory pathway or vomeronasal system (VNS), a group of forebrain nuclei indirectly innervated by the vomeronasal organ, relayed by the accessory olfactory bulb [2]. They receive and process olfactory information related to conspecific and heterospecific interactions [3]. Studies in rats and mice have shown that the PMv’s primary input and output targets are other components of the VNS located in the hypothalamus, amygdala, and bed nucleus of the stria terminalis [4–7]. Most of these nuclei are key components of neuroendocrine and behavioral circuitry and have already been found to be sexually dimorphic in rats [8–11].

The PMv expresses virtually all gonadal steroid nuclear receptors, with particularly dense levels of androgen receptor (AR) [12–14]. It also projects to the medial preoptic area, the anteroventral periventricular nucleus of the hypothalamus (AVPV), and the gonadotropin-releasing hormone (GnRH) neurons, the final neural output in the neuroendocrine reproductive axis [15–19]. The AVPV is larger in females [20] and a nodal hypothalamic site, mediating hormonal feedback fundamental for the luteinizing hormone (LH) surge at mid-cycle [21, 22].

The PMv expresses Fos immunoreactivity after mating, exposure to opposite- and same-sex odorants, and agonistic encounters in rats, mice, and hamsters [23–30]. Bilateral lesions of the PMv blocked the increase in LH release in female rats when they were presented with male urine [31]. Lesions of the PMv also block the increase in LH release in female rats after stimulation of the medial nucleus of the amygdala or the bed nucleus of the stria terminalis [31–33]. Male rats with bilateral-lesioned PMv showed a greater number of attacks and sustained an aggressive posture toward a non-lesioned same-sex cage mate [34]. More recent studies using not only lesions but also electrophysiology and genetically modified mice have shown that the PMv is a key place for integrating external and internal cues to generate socially relevant physiological and behavioral responses [35–38]. Akesson (1993) reported that the number of Substance P immunoreactive (SP-ir) neurons in the PMv was two times higher in male rats than in female rats. These neurons concentrate androgens, but their number does not respond to castration [39]. Yokosuka and co-workers (1997) reported sexual dimorphism in the expression of AR in the PMv in rats at 6 days of life. Indeed, virtually no AR-expressing neurons were observed in the PMv of female pups [40]. This seems to be reversed after puberty, since female adult rats presented AR-immunoreactive neurons in the PMv [41].

However, the sexual dimorphism of the structural organization of the PMv has never been studied. To achieve this goal, we used stereological techniques to estimate the volume of the PMv, the volume occupied by its neurons and neuropil, the density of the volume of its neurons, the mean of the neurons’ volume, and the number of neurons and compared them between males and females in estrus and diestrus.

## Methods

### Animals

To carry out this work, we used 21 5-month-old Wistar rats obtained from the Central Bioterium of the Biosciences Center of the Federal University of Rio Grande do Norte (Brazil). The animals were kept at constant temperature (22 °C), provided food and water without restrictions, under a 12:12 light-dark cycle, with the lights being turned on at 6:00 am and turned off at 6:00 pm. The animals were housed in a group of 4 by box, separated by sex, and with no contact with the opposite sex since weaning. The groups consisted of 8 males, 7 females in estrus, and 6 females in diestrus. The time of the cycle was detected via a vaginal smear. All procedures followed the standards for the use of experimental animals previously established by the Health Guide for the Care and Use of Laboratory Animals (2010) and by the Ethics Committee for Animal Use of the Federal University of Rio Grande do Norte (CEUA-UFRN) under the approval of CEUA-UFRN (protocol 021/2023; certificate 340.021/2023).

### Tissue preparation

The animals were anesthetized with ketamine (100 mg/kg) and xylazine (10 mg/kg) intraperitoneally and killed via transcardiac perfusion with 200 ml of saline solution (0.9%) to wash the systems, followed by 500 ml of paraformaldehyde fixative solution (4%) in 0.1 M phosphate buffer, pH 7.4.

After perfusion and decapitation, the animals’ heads were positioned in the stereotaxic apparatus for rodents. The bregma and lambda were leveled horizontally, and the cranial vaults were removed, exposing the brains. In the stereotaxic apparatus, the brains were sectioned into three blocks through two coronal sections, one at the level of the bregma and the other at the level of the lambda, using a scalpel blade attached to the arm of the stereotaxic apparatus. They were then removed, post-fixed overnight in the same fixative, and stored in sucrose solution (30%) at 4 °C for cryoprotection. The blocks containing the hypothalamus were cut in the frontal plane into 30-µm sections on a freezing microtome. Three series were collected in antifreeze solution and stored at − 20 °C. One series of samples from each animal was mounted and Nissl-stained, using Thionin. Thionin dyed the cytoplasm of the neurons, while it dyed just the nucleus of the glial cells, making it simple to differentiate one from another.

## Stereological analysis of the PMv

### Definition of the PMv

Our definition of the PMv was based on previous anatomical and neurochemical works focused on the PMv [4, 7, 23, 42, 43]. The PMv is a small nucleus of the basal hypothalamus surrounded by the arcuate nucleus, ventromedial nucleus of the hypothalamus, dorsal tuberomammillary nucleus, dorsal premammillary nucleus, fornix, and ventral border of the brain. It seems to have a more compacted group of rounded neurons in its ventral aspect, which is continuous throughout the nucleus, and a less compacted group of oval-to-fusiform neurons in the anterodorsomedial aspect. However, as the literature does not consider any anatomical division of the PMv, we treated it as a single nucleus.

### Reference volume (V_PMv_)

The total volume of the PMv was estimated based on the principle of Cavalieri [44, 45] in the same sections used for dissector counting. The sections were analyzed with a 5x objective and software-projected 3 × 3 virtual grid of points, and each point was associated with a known area. The volume was estimated via the following formula:

$$\:{V}_{PMv}:\:=\:\sum\:P\:.\:(a/p)\:.\:\stackrel{-}{t}\:.\:{ssf}^{-1}$$

where ∑P is the sum of the projected test point system interacting with the region; (a/p) is the area associated with each point (2500 µm^2^); $\:\:\stackrel{-}{t}\:$is the section thickness (30 μm); and $\:{ssf}^{-1}$ is the section sampling fraction (1/3).

To assess the reliability of the Cavalieri estimations, the Coefficient of Error (CE) was calculated via the method described by Gundersen and Jensen (1987) [46]. CE lower than 10% was considered acceptable.

Total number of PMv neurons (N_PMvneurons_).

The Optical Fractionator method [45, 47] was used to estimate the total number of neurons in the PMv. A 63x oil-immersed objective (1.4–0.6 aperture) was utilized, and estimations were made by the following formula:

$$\:{{\boldsymbol{N}}_{\boldsymbol{P}\boldsymbol{M}\boldsymbol{v}\boldsymbol{n}\boldsymbol{e}\boldsymbol{u}\boldsymbol{r}\boldsymbol{o}\boldsymbol{n}\boldsymbol{s}}:=\sum\:{\boldsymbol{Q}}^{-}\mathrm{\:.\:ss}{\mathrm{f}}^{\mathrm{-1}}\mathrm{\:.\:as}{\mathrm{f}}^{\mathrm{-1}}\mathrm{\:.\:}\mathrm{hs}{\mathrm{f}}^{\mathrm{-1}}}_{}$$

where Ʃ.${Q^ - }$. is the number of particles (in this case, PMv neurons) counted by the optical dissector probe; the optical dissector is associated with an unbiased counting frame (a(frame) = 2500 μm²). Ssf is the section’s sampling fraction (1/3). On the sampling sections, each counting frame was projected and spaced equidistantly in a systematic uniform random sampling (SURS) way, and its step length (distance between the projected counting frames), asf, is the area sampling fraction and is an inverse ratio between a (frame) and step length (16), and hsf is the height sampling fraction, which is a ratio between the section cut thickness and dissector height.

### Mean neuronal volume (Vn_PMvneurons_)

The mean body volume of PMv neurons was estimated via the Nucleator Method [45, 48], which is a local and direct estimator of particle volume. The method requires a uniquely defined subspace of the particle, e.g., the nucleus or the nucleolus of a cell, and the optical disector is used as a probe to sample the particles. In this study, the nucleator was computer-assisted using the 6 half-line nucleator probes available in the software Stereo Investigator^®^ MBF bioscience (version 11.09) and in the same reference sections used for total number estimation. The following formula was used to estimate the mean neuronal body volume:

$${\overline {v} _N} ~ = {\text{ }}\pi .{\text{ t}}.{\text{ }}\sum {\mathrm{ln}}^{{\mathrm{2}}}$$

Where ln^2^ is the distance from the center of the cell profile to the edge of the software-projected virtual six-line test system.

### Volume density of PMv neurons (Vv_PMv_)

On the basis of Delesse´s principle, the fractional volume of PMv occupied by neurons and their respective processes was determined by point counting in the same sections and employing the same number of points used for the Cavalieri estimate. A SURS of fields was elicited, and test points were randomly superimposed by computer-assisted Stereo Investigator^®^ MBF bioscience (version 11.09). We counted the total number of points falling within the PMv and the total number of points falling on neurons and their respective processes. The volume density was therefore estimated as follows [45]:

$${\text{Vv }}\left( \% \right)=\sum {\text{ PN}}_{{{\mathrm{PMv}}}} /{\text{ }}\sum {\text{ PT}}_{{{\mathrm{PMv}}}} \times {\mathrm{1}}00$$

### Total volume of PMv neurons (Vtot_PMv_)

The total volume of neurons (Vtot_PMv_) and their respective processes were indirectly estimated by multiplying their respective fractional volumes (Vv_PMv_) by the reference volume of PMv (V_PMv_) [49].

$${\mathrm{Vtot}}_{{{\mathrm{PMv}}}} = {\text{ Vv}}_{{{\mathrm{PMv}}.{\text{ VPMv}}}}$$

### Neuronal density

Through the PMv volume and total number of neurons, the number of neurons per mm³ was estimated via simple division for each case.

### Statistical analysis

Statistical analysis was performed using GraphPad Prism 8.0^®^ statistical software. Initially, the data were subjected to normality tests (Shapiro-Wilk). For groups that presented normal distribution and homoscedasticity of variances, the parametric Student’s t-test or one-way ANOVA test for independent samples was used, followed by Tukey’s post-hoc test for multiple comparisons. For groups that presented a non-normal distribution, the non-parametric Kruskal-Wallis test and Dunn’s post-hoc test were used for multiple comparisons. Differences between groups were considered significant when *p* < 0.05.

## Results

**Fig. 1:**
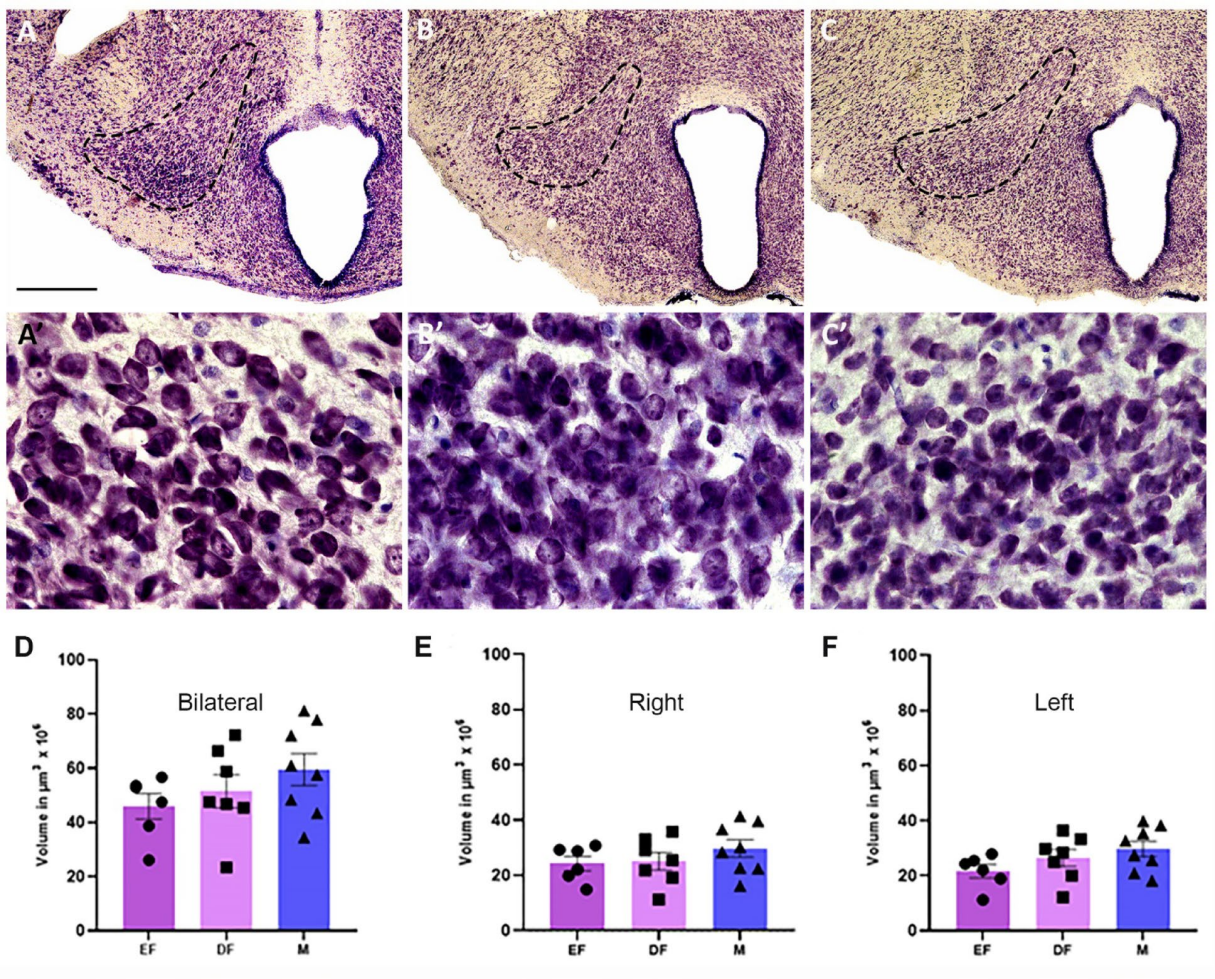
Anatomy and volume of the ventral premammillary nucleus (PMv). Photomicrographs of the basal hypothalamus highlighting the left PMv of a male (**A**), an estrus female (**B**), and a diestrus female (**C**), followed by a higher magnification photo of the center of each respective nucleus (A’, B’, and C’). Representative graphs of the estimated volume of the PMv bilaterally (**D**), in the right (**E**), and in the left (**F**) antimeres across the different groups: estrus female (EF), diestrus female (DF), and male (M). Vertical bars represent group means, and error bars indicate the standard error of the mean. Scale bar: 400 μm A, B, and C; 50 μm A’, B’, and C’

As described above, the PMv is a small, compactly organized group of neurons in the mediobasal hypothalamus. Rostrally, where the third ventricle is divided into the mamillary recess, it has a half-moon shape and is positioned laterally to the arcuate nucleus and ventromedially to the fornix. In its caudal half, it has an oval shape and is ventral to the dorsal premammillary nucleus and fornix. No obvious shape or location differences between the sexes were detected (Fig. 1).

### Volume of the PMv (V_PMv_)

The PMv volume data are compiled in Table 1 and presented in Fig. 1D-F.

The CE calculation was below 7% in both antimeres of all groups.

When summed bilaterally, the mean volume of the PMv in males was 59,49 ± 5,95 × 10^6^ µm^3^, in diestrus it was 51,49 ± 6,11 × 10^6^ µm^3^, and in estrus it was 45,9 ± 4,73 × 10^6^ µm^3^, with no statistically significant differences between the groups (one-way ANOVA, F_(2,18)_ = 1.396; *p* = 0.2730; estrus female vs. male *p* = 0.2540; diestrus female vs. male *p* = 0.5811). Although the mean PMv volume in males was approximately 22% larger than that in females, with the difference ranging from 15% to 30% across the estrus cycle (Fig. 1D), there was no statistical difference. Females in diestrus presented a PMv volume that was 12% greater than that in estrus, but multiple comparisons also revealed no statistically significant differences (*p* = 0.7911).

In both antimeres, the PMv of the male group presented a larger mean nuclear volume than both female groups did (Table 1; Fig. 1E and F). However, there was no statistically significant difference for the right (one-way ANOVA, F_(2,18)_ = 0.9439; *p* = 0.4075) or for the left antimeres (one-way ANOVA, F_(2,18)_ = 1.987; *p* = 0.1661).

There were no statistically significant differences when we compared antimeres in the same group (Student’s t-test, female estrus t_(10)_ = 0.7392; *p* = 0.4768; female diestrus t_(12)_ = 0.3018; *p* = 0.7680; and male t_(14)_ = 0.01983; *p* = 0.9845).

**Table 1:** Mean ± SEM of volume by group and antimere in the PMv (V_PMv_)

Group	Right antimere	Left antimere
Estrus female	24,26 ± 2,59 × 10^6^ µm^3^	21,64 ± 2,42 × 10^6^ µm^3^
Diestrus female	25,07 ± 3,20 × 10^6^ µm^3^	26,42 ± 3,12 × 10^6^ µm^3^
Male	29,70 ± 3,19 × 10^6^ µm^3^	29,78 ± 2,82 × 10^6^ µm^3^

### Volume density of neurons in the PMv (Vv_PMv_)

**Fig. 2:**
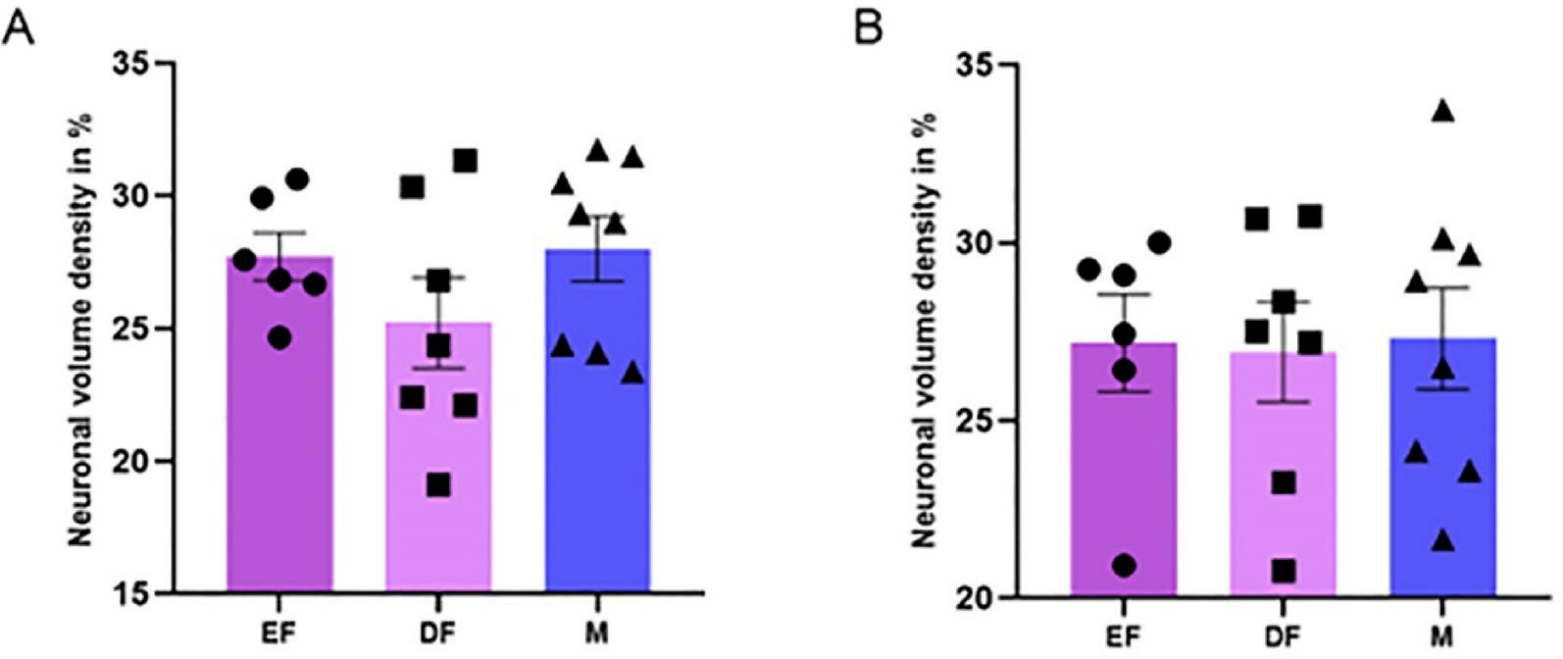
Representative graphs of the estimated neuronal volume density in the ventral premammillary nucleus (V_VPMv_) for the right (**A**) and left (**B**) antimeres across the different groups: estrus female (EF), diestrus female (DF), and male (M). Vertical bars represent group means, and error bars indicate the standard error of the mean

The data on the volume density of neurons are presented in Fig. 2. The neuronal bodies account for approximately 28% of the PMv volume in males, 26% in females in diestrus, and 27% in females in estrus. Therefore, there was no statistically significant difference for the right antimere (one-way ANOVA, F_(2,18)_ = 1.299; *p* = 0.2971). The same was true for the left antimere (one-way ANOVA, F_(2,18)_ = 0.01930; *p* = 0.9809).

### Total volume occupied by the neurons in the PMv (Vtot_PMv_)

**Fig. 3:**
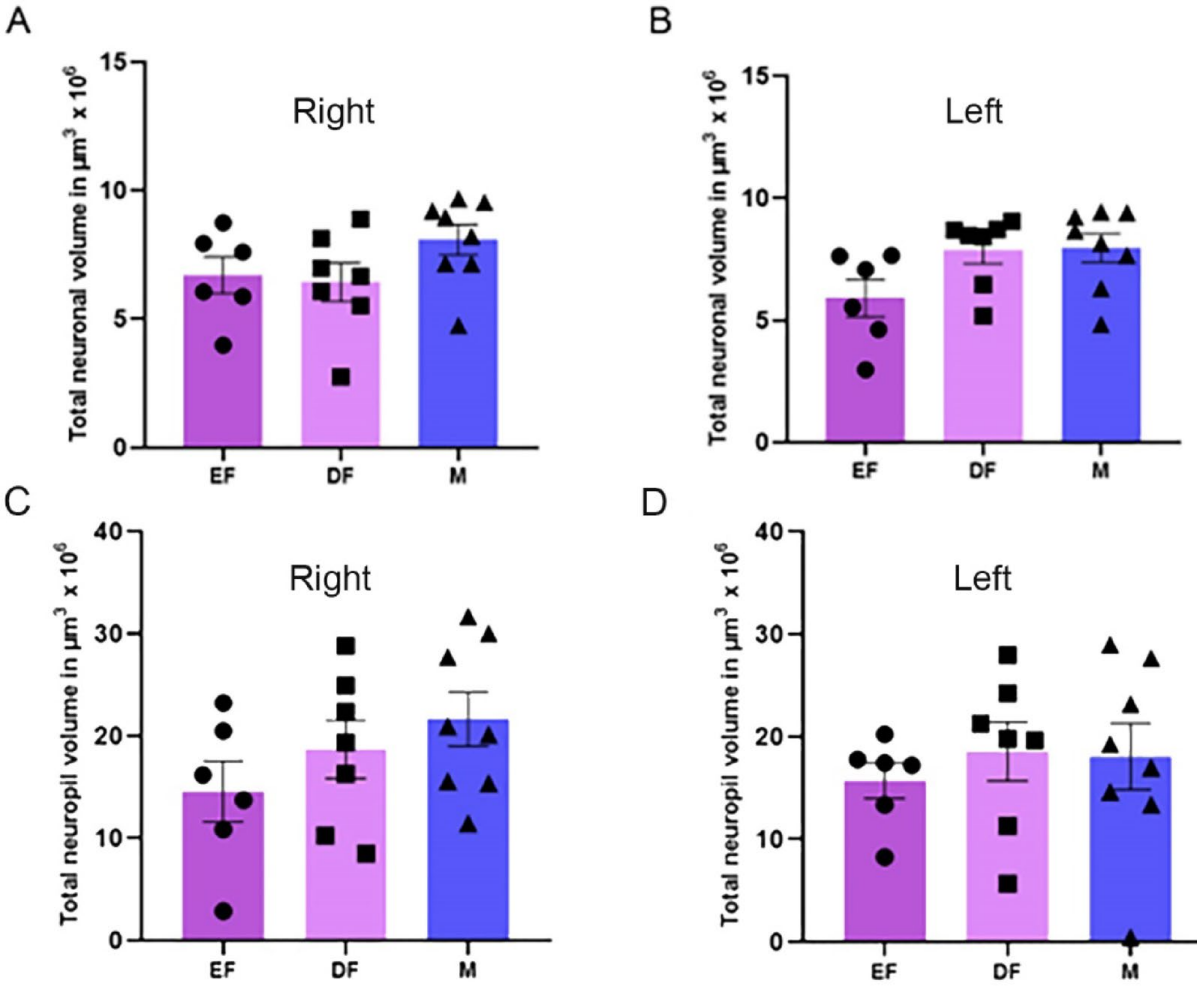
Representative graphs of the estimated total volume occupied by neurons in the ventral premammillary nucleus (Vtot_PMv_) and its neuropil for the right (**A** and **C**) and left (**B** and **D**) antimeres across the different groups: estrus female (EF), diestrus female (DF), and male (M). Vertical bars represent group means, and error bars indicate the standard error of the mean

The data on the total volume occupied by neurons are compiled in Table 2 and presented in Fig. 3A and B. Compared with the female group, the male group had a greater mean volume occupied by neurons, but the groups did not significantly differ (one-way ANOVA, F_(2,18)_ = 1.818; *p* = 0.1910). The groups in the left antimere showed a nonnormal distribution, so the analysis was performed with the Kruskal‒Wallis test, which also revealed a no statistically significant result (X^2^(2) = 5.482; *p* = 0.593).

**Table 2:** Mean ± SEM of the total volume occupied by neurons in the different groups and by antimere in the PMv (Vtot_PMv_)

Group	Right antimere	Left antimere
Estrus female	6,70 ± 0,71 × 10^6^ µm^3^	5,91 ± 0,77 × 10^6^ µm^3^
Diestrus female	6,43 ± 0,75 × 10^6^ µm^3^	7,86 ± 0,55 × 10^6^ µm^3^
Male	8,08 ± 0,59 × 10^6^ µm^3^	7,96 ± 0,58 × 10^6^ µm^3^

### Total volume occupied by the neuropil in the PMv

The data on the total volume occupied by the neuropil (estimated through subtraction of Vv_PMv_ from V_PMv)_ are compiled in Table 3 and presented in Fig. 3C and D. Compared with the female group, the male group presented a larger mean neuropil volume; however, the difference was not statistically significant for the right antimere (one-way ANOVA, F_(2,18)_ = 0.6807; *p* = 0.5188) or the left antimere (one-way ANOVA test, F_(2,18)_ = 1.545; *p* = 0.2404).

**Table 3:** Mean ± SEM of the total volume occupied by neuropil in the different groups and by antimere in the PMv

Group	Right antimere	Left antimere
Estrus female	17,56 ± 1,94 × 10^6^ µm^3^	15,73 ± 1,74 × 10^6^ µm^3^
Diestrus female	18,65 ± 2,83 × 10^6^ µm^3^	18,56 ± 2,88 × 10^6^ µm^3^
Male	21,62 ± 2,65 × 10^6^ µm^3^	21,83 ± 2,37 × 10^6^ µm^3^

### Mean somatic volume (Vn_PMvneurons_)

**Fig. 4:**
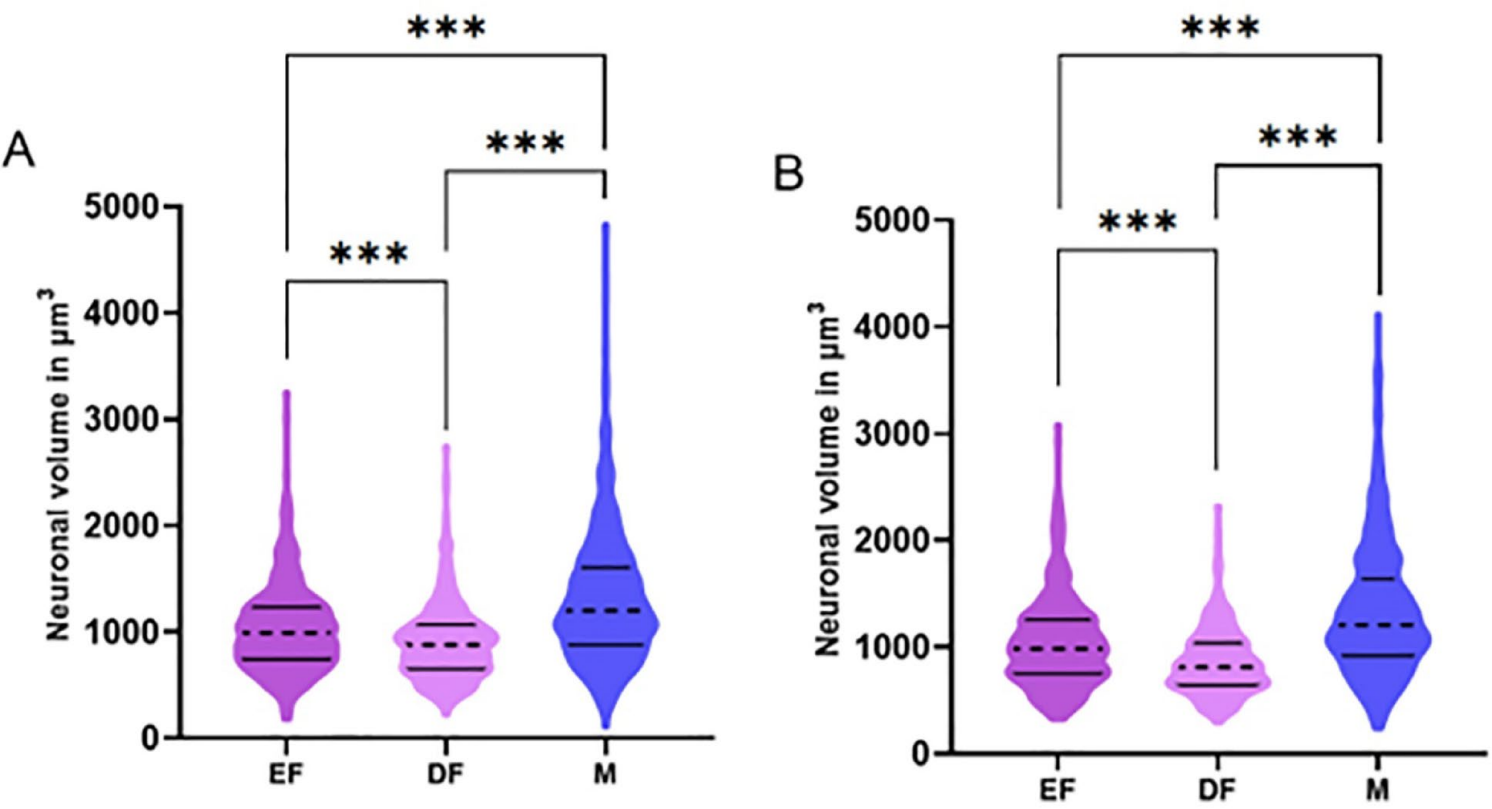
ViolinPlots of the average neuronal volume in the ventral premammillary nucleus (Vn_PMvneurons_) for the right (**A**) and left (**B**) antimeres across the different groups: estrus female (EF), diestrus female (DF), and male (M). The dashed line represents the median, and the solid parallel lines indicate the interquartile ranges. Comparisons were made using the Kruskal-Wallis test followed by Dunn’s post-hoc test. Significance levels are indicated by asterisks: ***p < 0,0001

For this parameter, the volume collected from each neuron was used as the sampling unit. The data from the right antimeres were *n* = 417 for the estrus female group, *n* = 473 for the diestrus female group, and *n* = 943 for the male group. The data from the left antimeres were *n* = 415 for the estrus female group, *n* = 574 for the diestrus female group, and *n* = 893 for the male group. The mean neuronal volume data are compiled in Table 4 and presented in Fig. 4.

The mean somatic volume of PMv neurons was 50% larger in males than in females in diestrus and 26% larger than in females in estrus. The mean somatic volume of the PMv was 18% greater in females in estrus than in those in diestrus.

A comparison via the Kruskal‒Wallis test revealed that for both antimeres, there was a statistically significant difference between the groups, with *p* < 0.0001 (right antimere X^2^(2) = 208.4; left antimere X^2^(2) = 302.4). According to the multiple Dunn’s post-hoc comparisons, in both antimere groups, the male group had a significantly greater mean neuronal volume than the estrus and diestrus female groups did (*p* < 0.0001). Compared with the diestrus female group, the estrus female group presented a greater mean neuronal volume (*p* < 0.0001) (Fig. 4).

**Table 4:** Mean ± SEM of average neuronal volume in the different groups and by antimere in the PMv (Vn_PMvneurons_)

Group	Right antimere	Left antimere
Estrus female	1036 ± 20,17µm^3#^	1045 ± 20,82µm^3#^
Diestrus female	897,7 ± 15,87µm^3^	861,7 ± 13,09µm^3^
Male	1302 ± 20,79µm^3^*^#^	1328 ± 20,08µm^3^*^#^

* *p* < 0,0001 compared to estrus females. # *p* < 0,0001 compared to diestrus females

### Total number of neurons (N_PMvneurons_)

**Fig. 5:**
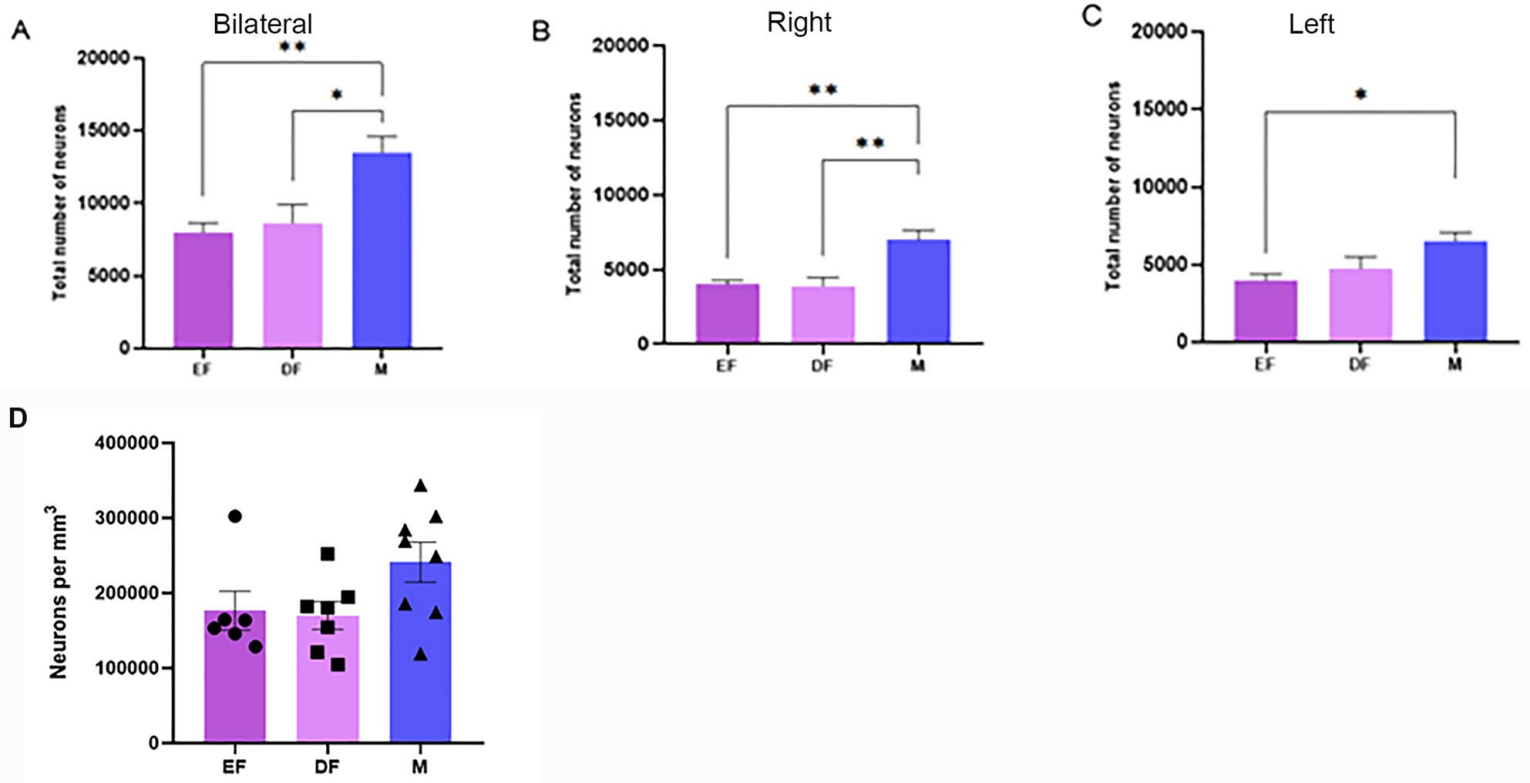
Representative graphs of the estimated number of neurons of the ventral premammillary nucleus (N_PMvneurons_) and its density across the different groups: estrus female (EF), diestrus female (DF), and male (M). Estimated number of neurons bilaterally (**A**), in the right (**B**), and in the left (**C**) antimeres. (**D**) Estimated number of neurons per mm³ in the ventral premammillary nucleus. Vertical bars represent group means, and error bars indicate the standard error of the mean. Comparisons were performed using one-way ANOVA followed by Tukey’s post-hoc test. Significance levels are indicated by asterisks: **p < 0.005, *p < 0.05

When summed bilaterally, male PMv had a mean of 13,500 neurons (Table 5; Fig. 5A), which was 69% greater than that of the females in estrus and 56% greater than that of the females in diestrus (one-way ANOVA, F_(2,18)_ = 7.903; *p* = 0.0034). Tukey post-hoc test revealed the following results: Estrus female vs. male (*p* < 0.0067); diestrus female vs. male (*p* = 0.0121). Compared with females in estrus, females in diestrus had a mean of 8% more neurons. This difference was not statistically significant (*p* = 0.9165).

**Table 5:** Mean estimate of the total bilateral number of neurons in the different groups in the PMv (N_PMvneurons_)

Group	Bilateral sum
Estrus female	7987
Diestrus female	8632
Male	13,500*^#^

* *p* < 0,05 compared to estrus females. # *p* < 0,05 compared to diestrus females

**Table 6:** Mean estimate of the total number of neurons in the different groups and by antimere in the PMv (N_PMvneurons)_

Group	Right antimere	Left antimere
Estrus female	4003	3984
Diestrus female	3892	4740
Male	6984*^#^	6516*

* *p* < 0,05 compared to estrus females. # *p* < 0,05 compared to diestrus females

Compared with the estrus and diestrus female groups, the male group presented a greater number of neurons in both antimeres (right antimere, F_(2,18)_ = 10.12; *p* < 0.0011; left antimere, F_(2,18)_ = 4.75; *p* < 0.022). The data is compiled in Table 6 and presented in Fig. 5B and C. In the multiple comparisons in the Tukey post-hoc test, the male group presented a higher total number of neurons in the right antimere when compared to the estrus female group (*p* < 0.0048) and the diestrus female group (*p* < 0.0025). In the left antimeres, the male group also presented a higher number of neurons when compared to the estrus female group (*p* < 0.0228). However, there was no statistically significant difference between the male and diestrus female groups (*p* = 0.1077). There was no statistically significant difference in the number of neurons in the right (*p* = 0.9903) or left (*p* = 0.6771) antimeres between the estrus and diestrus female groups.

### Neuronal density

The data are compiled in Fig. 5D. The male group had a mean neuronal density of 241,109 neurons/mm^3^. Compared with that of a diestrus female (169,975 neurons/mm^3^) and estrus female (176,418 neurons/mm^3^), it was approximately 42% and 37% denser, respectively. However, the groups did not differ statistically (one-way ANOVA, F_(2,18)=_2.819; *p* = 0.0861). In multiple comparisons, there were also no statistically significant differences (estrus female vs. diestrus female *p* = 0.9822; estrus female vs. male *p* = 0.1762; diestrus female vs. male *p* = 0.1087).

### Discussion

In mammals, males and females often display differences in a variety of endocrine, physiological, and behavioral traits. These differences are a product of differentiated neural mechanisms that coordinate internal status and environmental cues for appropriate behavioral and physiological responses [1]. Numerous brain areas, including the amygdala, hippocampus, bed nucleus of the stria terminalis, and several nuclei of the hypothalamus, have been demonstrated to be sexually dimorphic [11, 50–53]. Sexual dimorphism can be manifested in many ways, such as differences in cell morphology, neuronal size, neuronal number, axonal fiber projections, and the cellular expression of receptors or neurotransmitters.

### Stereological aspects

Using stereological techniques in the rat hypothalamus, we present the first unbiased analysis of the structural organization of the PMv in males and females. We also analyzed females at different stages of the estrus cycle.

The study revealed that the volume of the PMv did not exhibit statistically significant sexual dimorphism, with each antimere measuring approximately 0.026 mm³. Nevertheless, when the volumes of both antimeres were combined, the male PMv was approximately 22% larger than the female PMv. This difference fluctuated across the estrous cycle, with males exhibiting a 15% larger PMv than females during diestrus and a 30% larger PMv than females during estrus. Stereological analyses revealed significantly larger volumes in male rats for the medial preoptic nucleus (MPN), arcuate nucleus (Arc), and ventromedial nucleus of the hypothalamus (VMH), by 60%, 29%, and 25% larger, respectively [8–10]. Similarly, the different mean volumes occupied by the neuronal bodies and neuropil within the PMv did not reach statistical significance. The larger neuropil volume in males was identified as a critical factor in the established sexual dimorphism of the MPN and VMH [9, 10].

The data demonstrated sexual dimorphism in the estimated neuronal population of the PMv. The findings were statistically significant, with the male population exhibiting a 69% greater neuron count than the female population. This degree of sexual differentiation exceeded that observed in the MPN and Arc, which presented 28% and 15% higher neuron numbers in males, respectively [8, 9], whereas the VMH exhibited no such sexual dimorphism in the neuronal population (10). Our data is similar to that reported for the sexually dimorphic nucleus of the MPN (SDN), which exhibited 70% higher neuron counts in males than in females [9].

The neuronal density within the PMv was also assessed. The PMv of male rats exhibited a mean neuronal density of 241,109 neurons/mm^3^, whereas that of diestrus female rats was 169,975 neurons/mm^3^, and that of estrus female rats was 176,418 neurons/mm3. On the basis of data derived from prior stereological investigations in rats [8–10], the estimated neuronal density for the Arc was approximately 107,000 neurons/mm^3^ in males and 110,000 neurons/mm^3^ in females. In the MPN, the estimated neuronal density was 133,000 neurons/mm^3^ in males and 165,000 neurons/mm^3^ in females. For the VMH, the estimated densities were approximately 140,000 neurons/mm^3^ in males, 193,000 neurons/mm3 in diestrus females, and 167,000 neurons/mm^3^ in proestrus females. These findings indicate that the neuronal population in the PMv of males is denser than other hypothalamic nuclei, whereas the density in females appears to be similar.

While no prior studies have quantified the total neuronal population within the PMv, Akesson’s research (1993) demonstrated sexual dimorphism in the number of SP-ir neurons in the rat PMv. Specifically, the SP-ir neuron count was estimated to be 1,337 in male rats and approximately 600 in females by antimere [39]. Although the authors did not employ stereological techniques, these SP-ir neuron numbers appear reasonable, amounting to approximately 20% of the total PMv neurons in males and 15% in females.

Yokosuka and colleagues (1997) identified a sexually dimorphic population of AR-expressing neurons in the PMv of neonatal rats. While 6-day-old males presented a small number of AR-positive cells, female pups presented virtually no AR expression. This sexual disparity in AR levels within the PMv was abolished after puberty, as female rats began to express AR [41]. Additionally, researchers have reported no sex-based differences in the immunoreactivity of nitric oxide synthase, estrogen, and aromatase within the PMv of infant and adult rats [40, 41].

The volume of the PMv neurons exhibited sexual dimorphism, with males having larger PMv neurons than females. The sex-based differences were approximately 50% greater than those of females during diestrus and 26% greater than those of females during estrus. Similar findings were observed in other hypothalamic nuclei, where male rats presented larger neurons in the MPN, VMH, and Arc than females did [8–10].

The estrous phase influenced neuronal size, with PMv neurons being 18% larger in females during estrus than in diestrus. Previous research by Madeira and colleagues (2001) demonstrated that the VMH neurons were larger in female rats in proestrus than in diestrus. Proestrus is a phase characterized by high estrogen levels, which induces VMH neuron enlargement [54, 55]. In the present study, we analyzed PMv neurons during the diestrus and estrus phases. During the diestrus, estrogen and progesterone levels are low. During the estrus, which occurs after proestrus, estrogen levels decrease, whereas progesterone levels remain elevated. Ovariectomized female rats treated with estrogen and progesterone exhibited a larger medial preoptic area compared to ovariectomized or diestrus females [56]. These findings suggest that the high progesterone levels might be enough to keep larger PMv neurons during the estrus phase than during diestrus.

### Functional aspects

The PMv is part of the sexually dimorphic circuit, establishing massive bidirectional connections with sexually dimorphic nuclei. In other words, it receives information from these regions and sends substantial projections back to them, indicating a complex interaction that can modulate aggression and reproduction [57].

The PMv exhibits Fos immunoreactivity following mating or exposure to opposite-sex odors [23, 26, 29, 30]. Additionally, the PMv sends dense projections to the Arc and ventrolateral subdivision of the VMH (VMHvl), which are components of the reproductive neuroendocrine and behavioral circuitry, respectively [4, 6]. Furthermore, AVPV and GnRH neurons receive connections from the PMv [16, 18], suggesting the PMv’s capacity to directly or indirectly influence the secretion of sexual hormones. Importantly, bilateral lesions of the PMv were found to block the increase in LH release in ovariectomized, estrogen-primed female rats when presented with male urine or after stimulation of the medial nucleus of the amygdala or bed nucleus of the stria terminalis, areas known to increase LH in females [31]. Female rats with a lesioned PMv also exhibited temporary disruption of estrus, decreased numbers of GnRH neurons and Fos-expressing AVPV neurons during proestrus, blockade of the leptin-induced LH increase, reduced sexual receptivity on the night of estrus, and a significant absence of lordosis behavior in the presence of a male rat, thereby impacting the frequency of mounting and ejaculation in males [25, 58].

PMv neurons also interact with the Kisspeptin-Kiss1 system, which plays a role in the neuroendocrine regulation of GnRH release. During the proestrus phase in female rats, an increase in Kiss1 receptor mRNA within the AVPV is observed, triggering the LH surge [59]. Lesions of the PMv disrupt the typical physiological fluctuations of Kiss1 mRNA in the AVPV and periventricular nucleus, which are characteristic of the transition from proestrus to estrus [25].

The PMv neurons in rats and mice presented increased expression of Fos-ir after agonistic behaviors [28, 29]. Animals with lesioned PMv displayed an increased number of attacks and maintained an aggressive posture toward a same-sex cage mate with an intact PMv [34]. Studies using genetic and molecular tools in mice have further implicated the PMv in various forms of aggression, including aggression between conspecifics, which is predominantly observed in males, and maternal aggression, a behavior exclusive to females. A subset of PMv neurons expresses the dopamine transporter and are termed PMvDAT neurons. These PMvDAT neurons are glutamatergic and constitute between 25% and 34% of PMv neurons [60, 61]. In males, these neurons respond more intensely to odors from same-sex individuals than do those from females [60, 62]. The inhibition of PMvDAT neurons eliminated male responses to male odors and reduced aggression toward intruders without affecting reproductive behavior [60–62]. By separating a mouse population into aggressive and non-aggressive animals, Stagkourakis and colleagues (2018) reported that PMvDAT neurons are significantly more active in aggressive animals and that their stimulation promotes long-lasting aggression. PMvDAT neurons project to the VMHvl, which in turn sends projections back to the PMv, generating positive feedback since both are glutamatergic and are involved in determining social hierarchy [61].

Using genetic approaches, Itakura and colleagues (2022) identified a vomeronasal receptor responsive to the 53AF molecular weight fraction of male urine. Stimulation of Vmn2r53 by 53AF was sufficient to initiate aggression between conspecifics, and ablation of Vmn2r53 altered the response to male mouse urine but not to female odors [37]. Neurons in the PMv expressing the progesterone receptor (PMvPR) strongly respond to the presence of male conspecifics or their urine. These neurons receive input from the BST and MeA, are activated by 53AF, and project to the VMHvl. The inhibition of PMvPR neurons suppressed aggressive behavior in mice with prior aggressive experience, and their activation promoted aggressive behavior against male mice, even when they were castrated [37].

While less prevalent and less expressive than males, females can also display aggressive behaviors, particularly in specific situations, such as protecting their offspring. This behavior, known as maternal aggression, is driven by environmental cues and aims to safeguard offspring from potential threats [63, 64]. Elevated levels of Fos in the PMv of lactating rats have been observed following the expression of maternal aggression, and lesions to the PMv have been shown to reduce this behavior [65]. Maternal hormones such as prolactin and oxytocin stimulate PMvDAT neurons, transitioning these cells from a quiescent state to a hyperexcitable state during lactation. This heightened excitability is more pronounced in aggressive female mothers than in nonaggressive mothers or virgin females [66]. Stimulating PMvDAT neurons increases aggressive conduct and diminishes maternal behavior in lactating females. Conversely, inhibiting or genetically ablating PMvDAT neurons significantly reduces aggressive behavior in lactating females [66].

### Perspectives and significance

Differences in neuronal populations and their hormonal responsiveness may underlie sex-specific strategies in reproduction and aggression. Males may exhibit pronounced aggressive behaviors to secure mating opportunities, whereas females may engage in different strategies, prioritizing mate selection and offspring care. Understanding these differences could provide insights into evolutionary strategies and social dynamics within this species. Previous studies have highlighted the PMv as a crucial site for the integration of external environmental signals and internal physiological states, particularly in the context of social interactions, including mate selection, competition, and parental care [57].

While androgen-responsive neurons in the PMv are spread all over the nucleus, estrogen-responsive neurons are almost entirely confined to the ventrolateral aspect of the nucleus [41, 67]. This could mean that male rats not only have more and bigger neurons in the PMv, but also different neurons that respond to androgens, generating masculine physiology and behavior.

We also hypothesize that agonistic interactions between male rats may require the activation of a greater number of cells, as well as a larger neuronal volume, to accommodate increased metabolic activity, whereas in females, the differences during the estrous cycle might reflect the hormonal fluctuations, influencing the physiology and behavior.

### Conclusion

The current study revealed remarkable sex-based differences in the structural organization of the rat PMv. Specifically, the PMv of male rats contained more and larger neurons compared to female rats. However, these differences did not correspond to a significant volumetric disparity. Additionally, the volume of neurons exhibited dynamic changes throughout the estrous cycle. The findings of the present study reflect the potential sexually dimorphic functions of the PMv. It appears that the PMv plays a more pronounced role in aggressive behavior mechanisms in males, whereas in females, the PMv has a more complex involvement in the reproductive axis.

## Data Availability

No datasets were generated or analysed during the current study.
